# Structural Insights and Intermolecular Energy for Some Medium and Long-Chain Testosterone Esters

**DOI:** 10.3390/molecules28073097

**Published:** 2023-03-30

**Authors:** Alexandru Turza, Petru Pascuta, Liviu Mare, Gheorghe Borodi, Violeta Popescu

**Affiliations:** 1 National Institute For R&D of Isotopic and Molecular Technologies, 67-103 Donat, 400293 Cluj-Napoca, Romania; 2 Department of Physics and Chemistry, Technical University of Cluj-Napoca, 400114 Cluj-Napoca, Romania

**Keywords:** testosterone, ester, crystal structure, lattice energy, solubility

## Abstract

Testosterone (17β-Hydroxyandrost-4-en-3-one) is the primary male anabolic-androgenic steroid. The crystal structures of two medium and two long esterified forms of testosterone, including enanthate, cypionate, decanoate and undecanoate, were determined by X-ray single crystal diffraction. The samples were also characterized by powder X-ray diffraction, FT-IR spectroscopy and thermal analysis (DTA, TG). Crystal packings and supramolecular features were described. The analysis of structural features was accomplished by computational methods in terms of the type of intermolecular interactions, crystal energies and Hirshfeld surfaces analysis. From a pharmaceutical point of view, the solubility of compounds was investigated.

## 1. Introduction

Testosterone (17β-Hydroxyandrost-4-en-3-one) is chemically a cholesterol derivative and the primary naturally occurring anabolic steroid. It can be regarded as an androstane derivative and the primary male sex hormone. It fulfills an important role in the development and maintenance of the male reproductive system, promoting the appearance of secondary male characteristics, including increased lean muscle mass, bone mass, body hair growth [1], and prevents osteoporosis [2,3]. The hormone is also present in the body of women in much smaller quantities than in men, being associated with certain physiological roles such as sexual function, musculoskeletal, cardiovascular and vulvovaginal health and cognitive function [4]. As an androgen receptor agonist, it exerts the anabolic-androgenic properties which are common to all of its derivatives/analogues which belong to this family [5]. Medically, testosterone is used to relieve symptoms related to low testosterone in men, breast cancer in women and in hormone therapy for transgender men [6]. Targeting androgen receptors has been shown to have a positive overall impact on health and the state of well-being, and it triggers an increased protein synthesis in certain tissues which contain androgen receptors, among which are bones and muscle tissues [7]. Optimal testosterone levels in elderly men induce an overall positive impact on general health; they help to decrease body and visceral fat mass, help increase lean muscle mass, improve cholesterol levels, and enhance carbohydrate metabolism [8]. Anabolic agents such as testosterone and its analogues/derivatives are often used by athletes, weightlifters and bodybuilders in order to boost performance and aid muscle recovery [9]. It can also be used to relieve or treat protein breakdown in catabolic conditions [10]. It should be emphasized that testosterone is used illicitly by athletes and its use is prohibited by the World Anti-Doping Agency.

**Figure 1:**
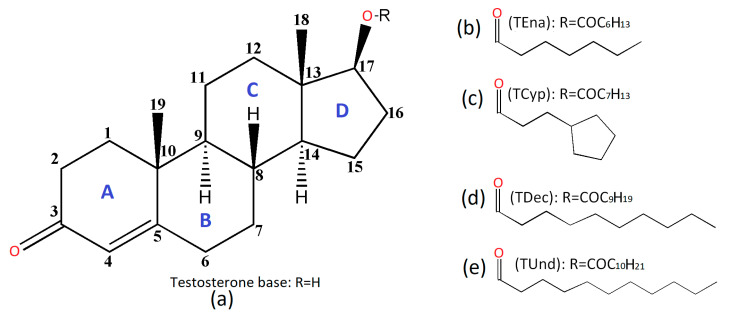
Chemical perspective of testosterone (17β-Hydroxyandrost-4-en-3-one), showing the backbone labeling system (**a**) and the studied testosterone-based prodrugs: enanthate (**b**), cypionate (**c**), decanoate (**d**), undecanoate (**e**).

Testosterone (Figure 1a) is characterized by a short half-life (a few hours), being subject to esterification, which leads to a considerable increase in the half-life depending on the length of the ester (of the carbon chain). Esterified forms offer the advantage of increasing the half-life when administered by intramuscular or subcutaneous injection, thus avoiding the need for daily administration [11]. Various esterified forms of testosterone show half-lives from less than 24 h for testosterone acetate [12] up to 36 days for testosterone undecanoate [13].

The current paper approaches and characterizes two medium and two long esterified testosterone prodrugs:

(i) Testosterone enanthate: Androst-4-en-17β-ol-3-one 17β-heptanoate, (TEna, Figure 1b);
(ii) Testosterone cypionate: Androst-4-en-17β-ol-3-one 17β-cyclopentylpropionate, (TCyp, Figure 1c);
(iii) Testosterone decanoate: Androst-4-en-17β-ol-3-one 17β-decanoate, (TDec, Figure 1d);
(iv) Testosterone undecanoate: Androst-4-en-17β-ol-3-one 17β-undecanoate, (TUnd, Figure 1e).

The numbering scheme for atoms and steroid backbone rings is presented in accordance with established notations for derivatives and analogues within this family. (Figure 1a) [14].

The literature reports the crystal structures of other shorter testosterone esters, along with their corresponding CCDC Refcodes, as follows: propionate (ZZZRCG01) [15], buciclate (ETEVII) [16], non-esterified form (TESTON10) [17], isocaproate, phenylpropionate and one propionate polymorph (with deposition numbers 2192707, 2192708 and 2192706) [18] and also acetate (TELYEO) [19]. Apart from testosterone esters, the crystal structures of other testosterone derivatives were also investigated [20,21,22]. This manuscript aims to analyze the structural features of various medium and long esters. Structural characterization was achieved by experimental methods including X-ray single crystal diffraction, powder X-ray diffraction, FT-IR spectroscopy and DTA/TG thermal analysis, and a quantitative evaluation of intermolecular interactions was completed by computational methods exploring lattice energies and intermolecular interactions in the solid state.

From a pharmaceutical perspective, it is known that certain pharmaceutical active ingredients are characterized by poor solubility in water, but at the same time they act as lipophilic compounds. Therefore, these compounds are better suited to dissolution in various lipid preparations [23]. Various agents such as hormones (progesterone, estradiol, testosterone esters and their derivatives), deoxycorticosterone and certain vitamins (K and E) are included in formulations which use oils as carriers [24,25]. The solubilities in various lipid-based mixtures were measured in light of this aspect. 

Considering the very large number of scientific papers about this hormone, we considered that it would be useful to supplement it with studies related to the structure, intermolecular interactions and solubility.

## 2. Results

### 2.1. Crystal Structures Analysis

In Figure S1 (Supplementary Materials), the match between the experimental powder X-ray diffraction patterns and simulated ones obtained based on the CIF files is presented. The data show a good overall agreement, which is indicative of purity, structural homogeneity and that the analyzed single crystals are representative of the entire samples. However, certain reflections are missing or barely visible. This feature can be attributed to a certain degree to the preferred orientation of crystallites in some samples (TDec and TUnd are pronounced in this regard).

Table 1 provides detailed single-crystal data and refinement information for the studied compounds.

#### 2.1.1. TEna (Testosterone Enanthate)

**Figure 2:**
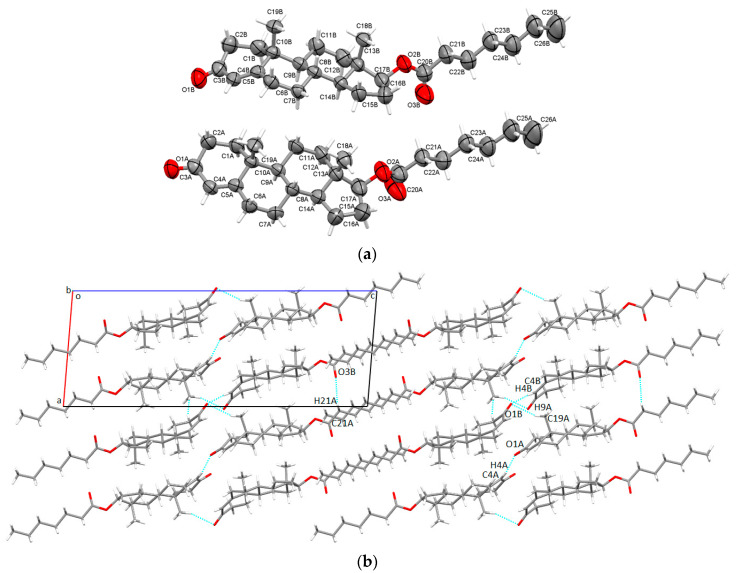
Asymmetric unit of Tena presenting non-hydrogen atoms at 50% probability level (**a**); overall packing viewed along b-axis (**b**).

The enanthate ester of testosterone was found to crystallize in the monoclinic P2_1_ space group, with two individual steroid molecules in the asymmetric unit (denoted with A and B suffixes, Figure 2a). They were bridged via C-H⋯O bonding between the O3B carbonyl acceptor of enanthate tail and CH_2_ donor (C21A-H21B⋯O3B interaction with an interaction magnitude of 45.1 kJ/mol). The values of interaction energies are further explored in detail in Section 2.3: Intermolecular energies evaluation. The ketone O1B oxygen was further building bifurcated C-H⋯O interactions, one to the CH_3_ group (C19A-H19B⋯O1B with an interaction energy of −29.3 kJ/mol) and one weakly linking the carbonyl O1B oxygen with the six membered A ring of molecule B (C4B-H4B⋯O1B, having a magnitude of −14.0 kJ/mol). On the other hand, O1A ketone oxygen was involved in the C4A-H4A⋯O1A hydrogen bond, which bound two A type molecules with an interaction magnitude of −14.6 kJ/mol. Overall packing shows that steroid molecules were assembled roughly diagonal with respect to the aoc plane (Figure 2b).

#### 2.1.2. TCyp (Testosterone Cypionate)

**Figure 3:**
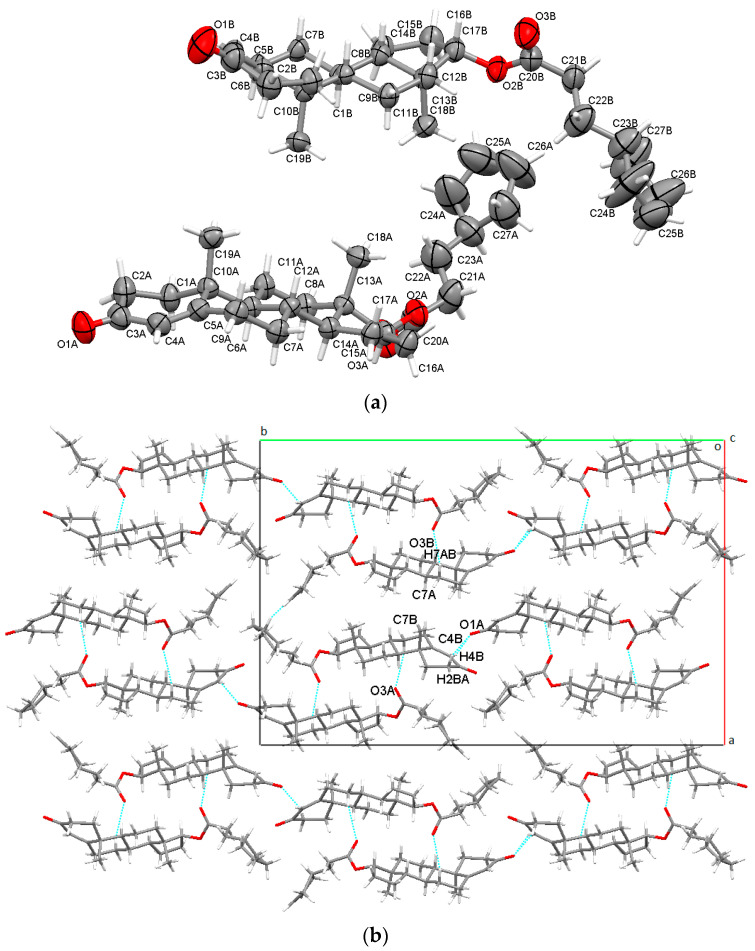
Asymmetric unit of TCyp presenting non-hydrogen atoms at 50% probability level (**a**); overall packing viewed along c-axis (**b**).

The cypionate ester of testosterone crystallized in the rare non-centrosymmetric P2_1_2_1_2 orthorhombic space group with two distinct molecules in the asymmetric unit (Figure 3a). O1A ketone oxygen was bounded in bifurcated hydrogen bonding with two neighboring A rings (C2B-H2BA⋯O1A with an energy of −20.3 kJ/mol and −14.8 kJ/mol for C4B-H4B⋯O1A). Further, both O3A and O3B carbonyl oxygens were involved in intermolecular interactions with other B steroid rings (C7A-H7AB⋯O3B was characterized by a high intercontact energy of −50.4 kJ/mol and weakly bounded energy of −18.8 kJ/mol for C7B-H8BB⋯O3A). The geometries of intermolecular interactions are detailed in Table S1 (Supplementary Materials), and in Figure 3b the crystal packing perspective seen along the c-axis is presented.

#### 2.1.3. TDec (Testosterone Decanoate)

**Figure 4:**
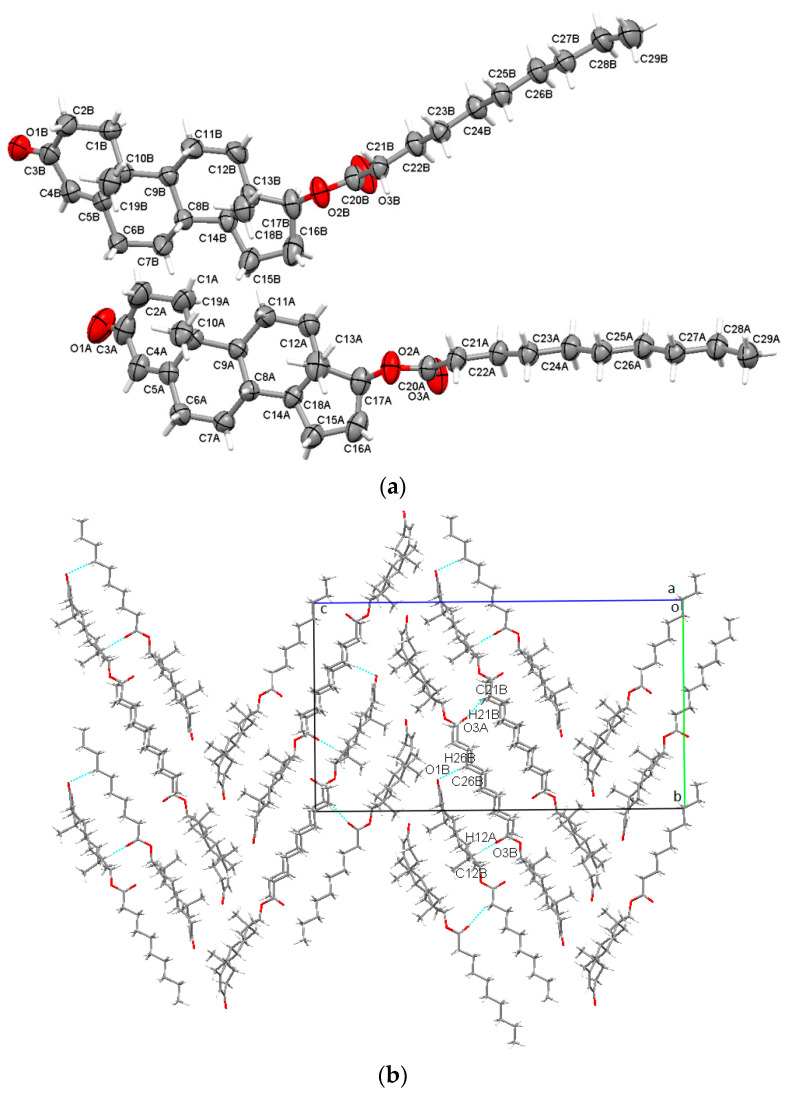
Asymmetric unit of TDec presenting non-hydrogen atoms at 50% probability level (**a**); overall packing viewed along a-axis (**b**).

The decanoate ester of testosterone is one of the longest available and was found to crystallize in the orthorhombic P2_1_2_1_2_1_ non-centrosymmetric space group, and consisted of two independent molecules in the asymmetric unit (Figure 4a). Crystal structure stability was held by a combination of C-H⋯O intermolecular bonding with O1B ketone oxygen, with O3A and O3B carbonyl oxygen playing the important roles of acceptors (a high value of interaction energy of −53.1kJ/mol for C21B-H21B⋯O3A; E_tot_ = −43.6 kJ/mol for C26B-H26B⋯O1B and C12B-H12A⋯O3B). The supramolecular arrangement seen along the a-axis shows that testosterone decanoate molecules adopt a W-shaped arrangement (Figure 4b). 

#### 2.1.4. TUnd (Testosterone Undecanoate) 

**Figure 5:**
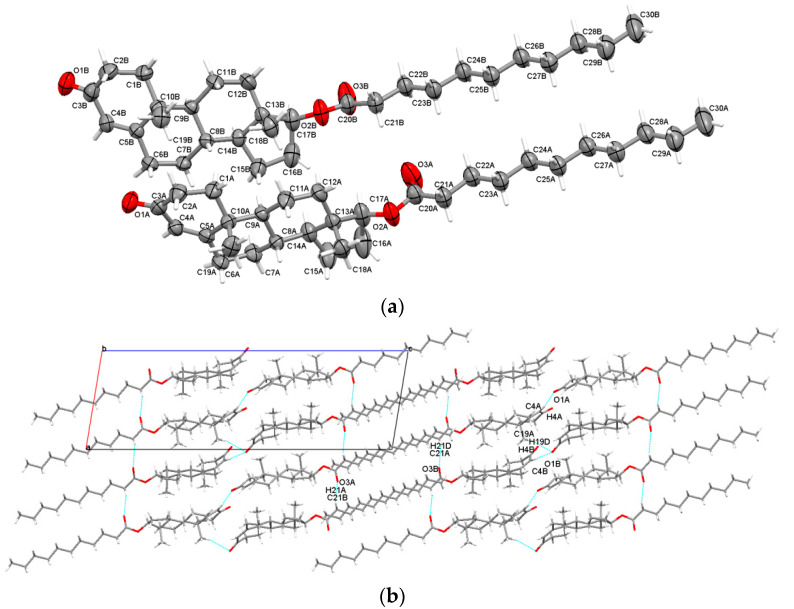
Asymmetric unit of TUnd presenting non-hydrogen atoms at 50% probability level (**a**); overall packing viewed along the b-axis (**b**).

This is the longest testosterone ester approached in the actual study. From a crystallographic point of view, it was found to crystallize in the same P2_1_ space group as the enanthate ester. In a manner similar to the previous esters, its asymmetric unit contains two individual steroid molecules (Figure 5a). By the use of both O3A and O3B carbonyl oxygens in the asymmetric unit, the steroid molecules adopt chain-like arrangements in the a-axis direction (the asymmetric unit molecules are tightly bounded with an energy of −60.8 kJ/mol for selected contact C21B-H21A⋯O3A, and for the second interaction in the a-axis direction C21A-H21D⋯O3B, E_tot_ = −51.1 kJ/mol). Once again, O1B oxygen from ketone serves as a hydrogen bonding acceptor, forming bifurcated interactions (C4B-H4B⋯O1B weakly connects a neighboring A ring, E_tot_ = −13.7 kJ/mol, while C19A-H19D⋯O1B with E_tot_ = −28.7 kJ/mol links the methyl CH_3_ of a neighboring A molecule). In a similar way, with testosterone enanthate the molecules are packed roughly diagonally with respect to the *aoc* plane (Figure 5b).

After analyzing the four crystal structures, the following conclusions can be drawn:

(i) All four esters crystallize in various non-centrosymmetric monoclinic and orthorhombic space groups;
(ii) Asymmetric units in all four crystals are characterized by two individual molecules, as opposed to the other short testosterone esters reported in the literature, such as acetate [19], propionate, isocaproate, phenylpropionate [18] and buciclate [16], which contain only one molecule;
(iii) Although C-H⋯O interactions participate in the formation of supramolecular 3D assemblies, their weight is quite small compared to dispersion effects (see the crystal energies analysis section);
(iv) C-H⋯O bonds are characterized by hydrogen⋯carbonyl donor-acceptor distances (Table S3, Supplementary Materials), which fall into the same range as other structures of the steroid class [26,27,28,29];
(v) The six membered A rings depict an intermediate sofa-half-chair conformation, B and C exhibit chair geometry, and the five membered D backbone rings display an intermediate envelope-half-chair geometry. The C17 methylated form [30] and other short esters have been shown to possess similar geometries [16,18].

### 2.2. Crystal Lattice Energies Evaluation

Total crystal energies and the breakdown in four energy components were evaluated using the atom–atom CLP model (Table 2). Further, the energies of the four medium and long esterified forms were compared with their non-esterified base form (TBas) and other short esters reported in the literature: acetate (TAce), propionate (TPro), isocaproate (Tiso) and phenylpropionate (TPhp) [18].

All nine crystals were characterized by dominant values of dispersion energies, the values increasing with the increase in ester length: TAce, being the shortest ester, shows a value of −126.1 kJ/mol, whereas TUnd, which possesses an eleven-carbon-longer ester chain, has a value of −175.4 kJ/mol. 

Regarding the Coulombian energy, because the structures lack strong hydrogen bondings, it contributes the least weight in the crystal packings, and their magnitudes are similar in all eight esters with an energy range of −15.5 kJ/mol for acetate (TAce) to −22.5 kJ/mol for enanthate (TEna). On the other hand, the Coulombic energy (−33.3 kJ/mol) in the non-esterified testosterone base form (TBas), which possesses classical hydroxyl-carbonyl O-H⋯O hydrogen bonding, shows an increased contribution to the total.

Polarization terms display similar values in six out of the eight esters (TAce, TPro, Tiso, TPhp, Tena, TCyp), but a slight increase in the longest two esters: −70.4 kJ/mol in decanoate (TDec) and −70.0 kJ/mol in undecanoate, showing that these two are slightly polarized.

Repulsion components do not show a trend related to ester length, and significant values are found in TBas and TEna.

With regard to total lattice energies, it can be noticed that crystal became more tightly bounded with the increase in ester length, from TBas −150.8 kJ/mol to Tund −220.7 kJ/mol, showing the highest stability of all nine. 

Previous reports in the literature show comparable results of other agents from the steroid family, where dispersion terms play the dominant role and the total energy increases with the increase in ester length [31,32,33,34].

### 2.3. Intermolecular Energies Evaluation

In order to have a complete perspective of the packing of the crystals, an evaluation of the intermolecular energies of the molecules for which the atoms are located at a smaller distance from each other than the sum of the Van der Waals radii was carried out. The attraction energies (electrostatic, polarization, dispersion), respectively, the repulsion energy and the total energy are presented in Table S4 (Supplementary Materials). 

The following conclusions result from Table S4:

Due to the long carbon tails, the molecules have an elongated shape. The interactions between parallel placed molecules are dominated by dispersion energy, due to the greater number of contacts and the small distances between them. For molecules that are placed end to end, the electrostatic interaction energy becomes important.

(i) Since the crystalline structures do not possess classic strong hydrogen bonds but C-H⋯O interactions, the electrostatic terms have small values in all cases;
(ii) The values of the polarization energies are very low, which indicates that the molecules are not polarized;
(iii) The values of the total energies are relatively low and vary in a wide range (−10.4 to −60.8 kJ/mol) due to the different orientations of the molecules relative to each other; between the neighboring molecules, there are rather weak interactions due to the lack of strong hydrogen bonds;
(iv) Table S4 (Supplementary Materials) shows that the dispersion energy is dominant.

### 2.4. Hirshfeld Surfaces and Fingerprint Plots Analysis

Molecular Hirshfeld surfaces of investigated crystals were generated via d_norm_ and compared to each other (Figure S2, Supplementary Materials). Based on the fact that the asymmetric units of all esters are comprised by two individual molecules, they required treating separately. 

Intermolecular contacts, which are shorter than the sum of van der Waals radii, were exemplified by arrows and listed in Table S1 (Supporting Information). Based on color coding (red, white, blue), the surfaces can be explained as follows: red dots denote strong intermolecular interactions with distances shorter than the sum of vdW radii, white patches represent the intermolecular contacts close to the sum of vdW radii, while weak interactions with longer distances are illustrated in blue. The fingerprint plots for each crystal, which are related to the 2D representation of the Hirshfeld surfaces, were generated and analyzed as well (Figure S3, Supplementary Materials).

Analyzing the Hirshfeld diagrams and fingerprint plots, certain structural features can be summarized:

(i) Fingerprint plots of esterified forms (Figure S3, Supporting Information) are asymmetric, which is characteristic of crystals with two or more molecules in the asymmetric unit and appears as a result of a distinct molecular environment in crystal;
(ii) The plots are illustrating H⋯O/O⋯H spikes, which denote the existence of C-H⋯O hydrogen bonds, but for TDec the H⋯O/O⋯H spikes are less protruding as a consequence of longer distances in the C-H⋯O bonds, which are closer to the sum of vdW radii;
(iii) A quantitative breakdown of fingerprint diagrams (Table S2, Supplementary Materials) shows the similarities in individual contributions in all four esters, with the highest percentage in H⋯H contacts, followed by a medium percentage of O⋯H/H⋯O intercontacts and a smaller one for C⋯H/H⋯C, respectively;
(iv) Based on the large percentages of H⋯H contacts for all crystals (fingerprint breakdown in Table S2, Supplementary Materials) corroborated crystal and intermolecular energies (Table 2 and Table S4 from Supplementary Materials) validate that dispersion components play the major role in overall packing.

### 2.5. Solubility Evaluation

**Figure 6:**
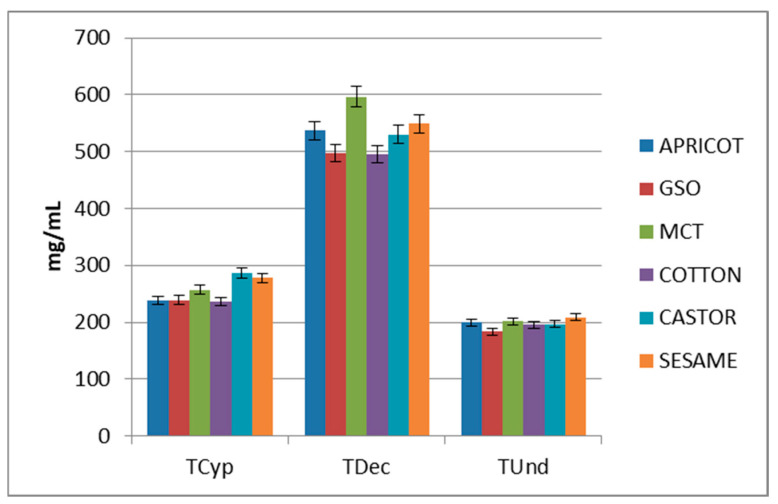
Graphical representations of esters solubility (standard deviation is represented by error bars)**.**

The solubility measurements are represented graphically in Figure 6 and listed in Table S3 (Supporting information). Abbreviations were used in Figure 6: MCT for medium-chain triglyceride, and GSO for grape seed oil. Unfortunately, the evaluation of the solubility of the enanthate ester was not possible because the solutions became transparent, gelatinous mixtures. Analyzing the values, it can be noted that there is no correlation between solubility and the length of the ester. The shortest ester (TCyp) showed an average solubility of roughly 255 mg/mL, the longest one (TUnd) was characterized by the lowest solubility of roughly 197 mg/mL, and a considerably greater solubility of about 2.5–3 fold greater (534 mg/mL) was recorded in the case of TDec.

What is worth noting is the fact that all six mixtures have similar solubilities.

The solubilities of some short esters have been previously reported, averaging 110 mg/mL for the acetate ester, 175.5 mg/mL for the propionate, 139 mg/mL for the phenylpropionate and 447 mg/mL for the isocaproate [18]. These show lower solubilities compared to long esters, but are comparable in orders of magnitude.

The solubility in castor oil of hydroxyprogesterone caproate polymorphs was previously reported in the literature as being 278 mg/mL and 301 mg/mL, respectively, at a temperature of 20° [35], which are comparable to the prodrugs investigated.

### 2.6. FT-IR Spectroscopy Analysis 

The IR spectra of esters are shown and compared in Figure S4 (Supplementary Materials). 

As can be seen from this figure, the absorption bands that appear in the 2800–3050 cm^−1^ wavenumbers region are similar and can be attributed to the symmetric and asymmetric C-H stretching within the CH, CH_2_, CH_3_ functional groups.

The two strong absorption bands that appear between wavenumbers 1670–1736 cm^−1^ are due to the carbonyl C=O groups present in the ketone and ester groups. At lower wavenumbers included in the 1611–1617 cm^−1^ range, sharp but less intense peaks are observed, which are attributed to C=C stretching of the carbons within the steroid A rings. In the fingerprint area (below 1500 cm^−1^), the spectra for TEna, TDec, TUnd are similar because only the number of carbon atoms in the tails differentiates them, while TCyp shows differences due to the presence of the cyclopentyl ring.

### 2.7. DTA/TG Analysis

DTA/TG curves of analyzed compounds are shown in Figure S5 (Supporting Information). Small endothermic peaks appear at (42 °C for Tena), (97 °C for TCyp), (49 °C for TDec) and (65 °C for TUnd), and are assigned to the melting points of the compounds. It should be mentioned that TEna has the lowest melting point, and in the solubility evolution process it became gelatinous even though it was assessed at 14 °C. 

With the increase in temperature, no thermal events occurred until the onset temperature of 320 °C for long esters (TDec, TUnd) and up to 350 °C for medium esters (TEna, TCyp). 

Further, two broad exothermic peaks were recorded. The first peak had its maximum at the following temperatures: (381 °C, TEna), (393 °C, TCyp), (378 °C, TDec) and (387 °C, TUnd). This peak can be attributed to the process of degradation and oxidation of organic matter, accompanied by a significant loss of mass. The second exothermic peak, with maxima (544 °C, TEna), (537 °C, TCyp), (540 °C, TDec), (540 °C, TUnd), can be associated with the decomposition and oxidation of the remaining material simultaneously with a less significant loss of mass. Similar thermal behavior has been reported for other active pharmaceutical ingredients such as riboflavin and norfloxacin [36].

## 3. Materials and Methods

### 3.1. Materials and Recrystallization Experiments

Crystalline powders of esters for scientific research purposes were received from Wuhan Shu Mai Technology Co. (Wuhan, China), while the solvents were received from Merck. Various oils of USP grade were received from Sigma-Aldrich (St. Louis, MO, USA), Tex Lab supply and Med Lab supply (USA).

Good quality single crystals for X-ray data collection were successfully grown in alcohols: isopropilic alcohol (TEna), or mixtures such as methanol–ethanol mixture (TDec, TUnd). The exception is TCyp, which was recrystallized in hexane solution.

### 3.2. Powder X-ray Diffraction 

The diffraction patterns were recorded with a Bruker D8 Advance diffractometer with the X-ray tube operating at 40 kV and 40 mA. It was equipped with a germanium (1 1 1) monochromator used to obtain only the CuKα1 radiation, and a LINXEYE detector. X-ray diffraction patterns for Tena and TUnd were recorded in the 2–40° range, and for TCyp and TDec they were recorded in the 3–40° (2θ) range using the DIFFRAC plus XRD Commander Program and a scanning speed of 0.02°/s.

### 3.3. Single Crystal X-ray Diffraction and Structures Refinement

Single crystal X-ray diffraction data were collected using a SuperNova diffractometer equipped with dual micro-sources (Cu and Mo) with the X-ray tube operating at 50 kV and 0.8 mA. The collection, reduction and correction of Lorentz, polarization and absorption effects were completed with CrysAlis PRO software [37]. The crystal structures of TCyp and TDec were solved with direct methods by SHELXS [38], TEna with SHELXD [38] and TUnd with SHELXT [39]. All were further refined with Least Squares minimization SHELXL [40], all mentioned programs being included in the Olex2 software [41]. 

A riding model was considered for hydrogen atoms treatment: the isotropic displacement parameter Uiso(H) = 1.2Ueq(C) for ternary CH groups [C-H = 0.93 Å], secondary CH_2_ groups [C-H = 0.97 Å] and 1.5Ueq(C) for all methyl CH_3_ groups [C-H = 0.96 Å].

### 3.4. Computational Methods

Computational results were achieved based on the fractional coordinates of atoms in the unit cell, determined by single crystal X-ray diffraction analysis. C-H bond distances for CH, CH_2_ and CH_3_ groups were normalized to C-H = 1.083 Å.

The crystal energies were calculated using the atom–atom potential Coulomb–London–Pauli (CLP) included in the CLP-Pixel package [42]. The method implies the evaluation of total lattice energies and the breakdown in four terms: Coulombic, polarization, and dispersion, which are attractive, and the fourth repulsive term.

CrystalExplorer software [43] was used to calculate the magnitudes and nature of intermolecular interaction energies. The interactions with distances shorter than the sum of van der Waals radii were computed as pairwise and imply the summation of four energy components: electrostatic (E_ele_), polarization (E_pol_), dispersion (E_dis_) and exchange repulsion term (E_rep_) [44]. The B3LYP/6-31G(d,p) exchange correlation functional and basis set was used with the scale factors k_ele_ = 1.019, k_pol_ = 0.651, k_disp_ = 0.901, and k_rep_ = 0.811.

The d_norm_ function was used to generate the Hirshfeld surfaces and fingerprint plots in CrystalExplorer software [43].

### 3.5. Solubility Evaluation

The esters’ solubility (mg/mL) was evaluated in mixtures which included different solvents and organic oils, such as medium-chain triglyceride (MCT), apricot, grape seed (GSO), castor, cottonseed, castor and sesame oil. 

The solutions contained mixtures of benzyl alcohol, benzyl benzoate and oil, with a volumetric ratio of 2% benzyl alcohol, 20% benzyl benzoate and 78% oil. Numerous pharmaceuticals of various lipophilic agents (various steroids were included) use benzyl benzoate as a co-solvent/solubilizer; benzyl alcohol behaves as well as a solvent and inhibits the microbial growth, and meanwhile the oils act as carriers.

The evaluation of solubility was completed in successive steps at an ambient temperature of 14 °C by the addition of small quantities of raw compounds (roughly 2–3 mg per step), while the solutions were stirred for several hours until completely dissolved. In certain cases, an excess of raw samples remained undissolved (in suspension) and small quantities of mixtures were added, ensuring that the resulting solutions became homogenous, clear and transparent. Three measurements were taken to improve accuracy, and their average value was used.

### 3.6. FT-IR Spectroscopy

FT-IR spectra of the samples were obtained using a Jasco 6200 FT-IR spectrometer, with 256 scans and a resolution 4 cm^−1^, in the spectral range 4000–400 cm^−1^. The samples were pre-prepared in the form of KBr disks with the pellet technique.

### 3.7. Differential Thermal Analysis (DTA) and Thermogravimetric Analysis (TG)

DTA/TGA measurements were performed with a simultaneous thermogravimetric and differential thermal analyzer Shimadzu DTG-60H. The samples were heated with a heating rate of 10°C/min, using an alumina sample cell (diameter 5.8 × 2.5 mm^2^) under a dry nitrogen purge (70 mL/min).

## 4. Conclusions

The crystal structures of four medium and long testosterone-based esters, including enanthate, cypionate, decanoate and undecanoate, were determined and reported. They were shown to belong to non-centrosymmetric monoclinic and orthorhombic space groups. The skeleton rings’ geometries were found to be sofa-half-chair in A rings and chair conformation for B and C rings, while five membered D rings depicted envelope geometry. Supramolecular arrangements were driven by dominant dispersion components, and the hydrogen⋯carbonyl C-H⋯O interactions played a lesser role in crystal stability. The solubility was evaluated, and the undecanoate form had the lowest solubility, followed by cypionate, while the decanoate form had a considerably higher solubility of roughly two- to threefold.

## Figures and Tables

**Table 1:** Crystal structures and refinement data of investigated compounds.

Identification Code	TEna	TCyp	TDec	TUnd
Empirical formula	C_26_H_40_O_3_	C_27_H_40_O_3_	C_29_H_46_O_3_	C_30_H_48_O_3_
Formula weight	400.58	411.58	442.66	456.68
Temperature/K	293(2)	293(2)	293(2)	293(2)
Crystal system	monoclinic	orthorhombic	orthorhombic	monoclinic
Space group	P2_1_	P2_1_2_1_2	P2_1_2_1_2_1_	P2_1_
a/Å	10.5644(6)	20.9920(6)	8.3367(3)	10.5820(2)
b/Å	8.2875(6)	32.0578(8)	19.0481(6)	8.23950(10)
c/Å	27.6918(15)	7.2292(2)	33.7169(12)	32.3890(4)
α/°	90	90	90	90
β/°	94.652(5)	90	90	99.3530(10)
γ/°	90	90	90	90
Volume/Å^3^	2416.5(3)	4864.9(2)	5354.2(3)	2786.47(7)
Z	4	8	8	4
ρcalc g/cm^3^	1.101	1.124	1.098	1.089
μ/mm^−1^	0.542	0.553	0.531	0.523
F(000)	880.0	1800.0	1952.0	1008.0
Radiation	CuKα (λ = 1.54184)	CuKα (λ = 1.54184)	CuKα (λ = 1.54184)	CuKα (λ = 1.54184)
2Θ range/°	6.404 to 141.12	6.938 to 145.1	7.002 to 148.164	8.3 to 141.722
Index ranges	−12 ≤ h ≤ 12, −10 ≤ k ≤ 9, −33 ≤ l ≤ 26	−25 ≤ h ≤ 25, −38 ≤ k ≤ 36, −7 ≤ l ≤ 8	−8 ≤ h ≤ 10, −23 ≤ k ≤ 23, −41 ≤ l ≤ 41	−12 ≤ h ≤ 12, −10 ≤ k ≤ 9, −39 ≤ l ≤ 39
Reflections collected	15,913	32,749	75,177	40,701
Independent reflections	8094 [R_int_ = 0.0422, R_sigma_ = 0.0471]	9217 [R_int_ = 0.0620, R_sigma_ = 0.0556]	10,249 [R_int_ = 0.1805, R_sigma_ = 0.0748]	10,192 [R_int_ = 0.0333, R_sigma_ = 0.0213]
Data/restraints/parameters	8094/1/529	9217/0/545	10,249/0/583	10,192/1/601
Goodness-of-fit on F2	1.001	1.031	1.085	1.044
Final R indexes [I>=2σ (I)]	R_1_ = 0.0549, wR_2_ = 0.1372	R_1_ = 0.0706, wR_2_ = 0.1833	R_1_ = 0.0938, wR_2_ = 0.1762	R_1_ = 0.0428, wR_2_ = 0.1141
Final R indexes [all data]	R_1_ = 0.0896, wR_2_ = 0.1652	R_1_ = 0.1199, wR_2_ = 0.2265	R_1_ = 0.1406, wR_2_ = 0.2100	R_1_ = 0.0462, wR_2_ = 0.1186
Largest diff. peak/hole/e Å^−3^	0.15/−0.18	0.25/−0.17	0.16/−0.22	0.17/−0.21
Flack parameter	−0.21(17)	0.04(18)	−0.02(16)	−0.09(8)

**Table 2:** Crystal lattice energies [18].

Structure	Molar Mass g/mol	E_coul_ (kJ/mol)	E_pol_ (kJ/mol)	E_disp_ (kJ/mol)	E_rep_ (kJ/mol)	E_latt_ (kJ/mol)
TBas	288.43	−33/3	−47.5	−130.9	60.9	−150.8
TAce	330.46	−15.5	−55.0	−126.1	37.1	−159.5
TPro	344.49	−18.3	−55.5	−126.4	33.9	−166.3
TIso	386.57	−21.3	−57.7	−141.9	52.7	−168.2
TPhp	420.59	−19.3	−52.3	−149.0	36.1	−184.5
TEna	400.60	−22.5	−60.4	−157.4	64.6	−175.7
TCyp	412.61	−17.6	−58.5	−142.8	37.0	−181.9
TDec	442.68	−17.4	−70.4	−167.3	40.1	−215.1
TUnd	456.71	−19.6	−73.0	−175.4	47.3	−220.7

E**_coul_**: Coulombic energy; E_pol_: polarization energy; E_disp_: dispersion energy; E_rep_: repulsion energy; E_latt_: total crystal lattice energy.

## Data Availability

CIF files of studied crystals were deposited with the Cambridge Crystallographic Data Centre with the following deposition numbers: 2233487 (TEna); 2233486 (TCyp); 2233488 (TDec); 2233489 (TUnd). Copies can be obtained free of charge on written application to CCDC, 12 Union Road, Cambridge, CB2 1EZ, UK (fax: +44-1223-336033); on request via e-mail to deposit@ccdc.cam.uk; or by access to http://www.ccdc.cam.ac.uk (accessed on 20 February 2023).

## Supplementary Materials

The following supporting information can be downloaded at: https://www.mdpi.com/article/10.3390/molecules28073097/s1, Figure S1: Powder X-ray diffraction patterns comparison Experimental and simulated XRPD: TEna (a), TCyp (b), TDec (c), TUnd (d); Figure S2: Hirshfeld surfaces mapped with d_norm_ ilustrating the contacts referred in Table S1. Surfaces were represented with the clour scale in the ranges as follows: TEna (a) −022 (red) to 174 (blue), −0.19 (red) to 1.93 (blue) for TCyp, −0.09 (red) to 1.68 (blue) for TDec, −0.20 (red) to 1.73 (blue) for TUnd; Figure S3: Fingerprint diagrams of analysed crystals; Table S1: Hydrogen bond geometry for studied crystals (Å, ^o^); Table S2: Contributions to the Hirshfeld surfaces for various intercontacts; Table S3: Solubility of esters in various mixtures; Table S4: Nature and magnitudes of intermolecular interaction energies for selected contacts (kJ/mol); Figure S4: FT-IR spectra of analyzed esters; Figure S5: DTA/TG curves of analyzed compounds
