# Supplementary material for: Structural Insights and Intermolecular Energy for Some Medium and Long-Chain Testosterone Esters
Source: Molecules. 2023 Mar 30;28(7):3097. doi: 10.3390/molecules28073097 (PMC10096163; doi:10.3390/molecules28073097)
Supplement: Supplementary file 1 [file molecules-28-03097-s001.zip › molecules-2266610-supplementary.pdf]

Supporting information

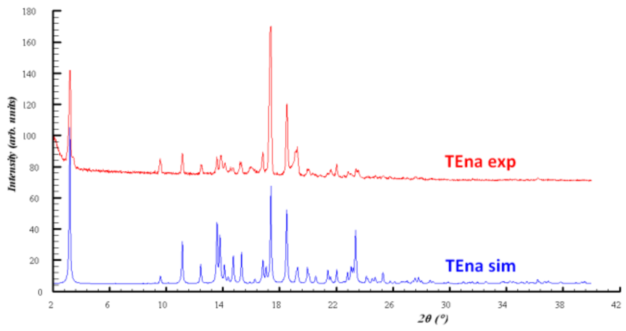

(a)

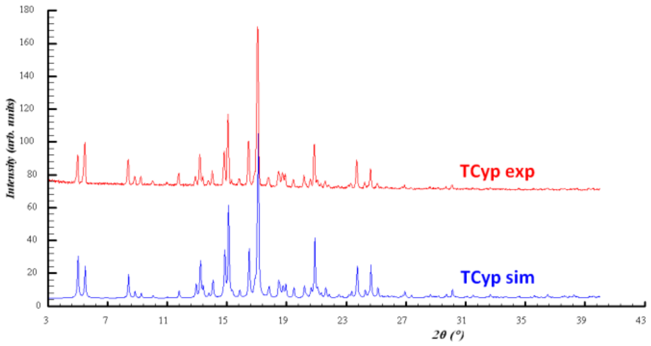

(b)

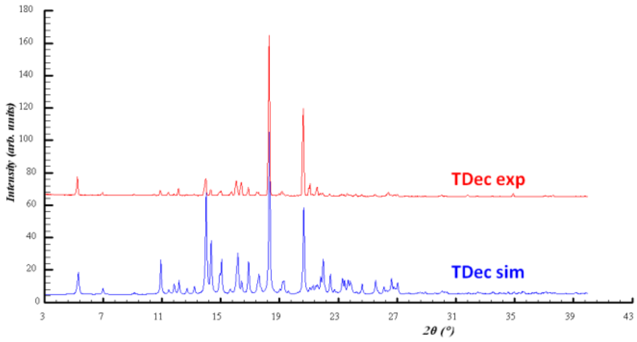

(c)

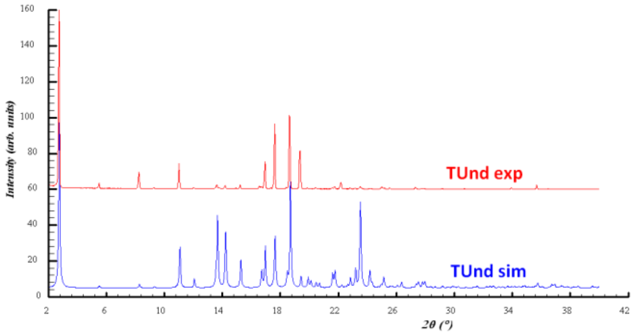

(d)

Figure S1. Powder X-Ray diffraction patterns comparison Experimental and simulated XRPD: TEna (a), TCyp (b), TDec (c), TUnd (d)

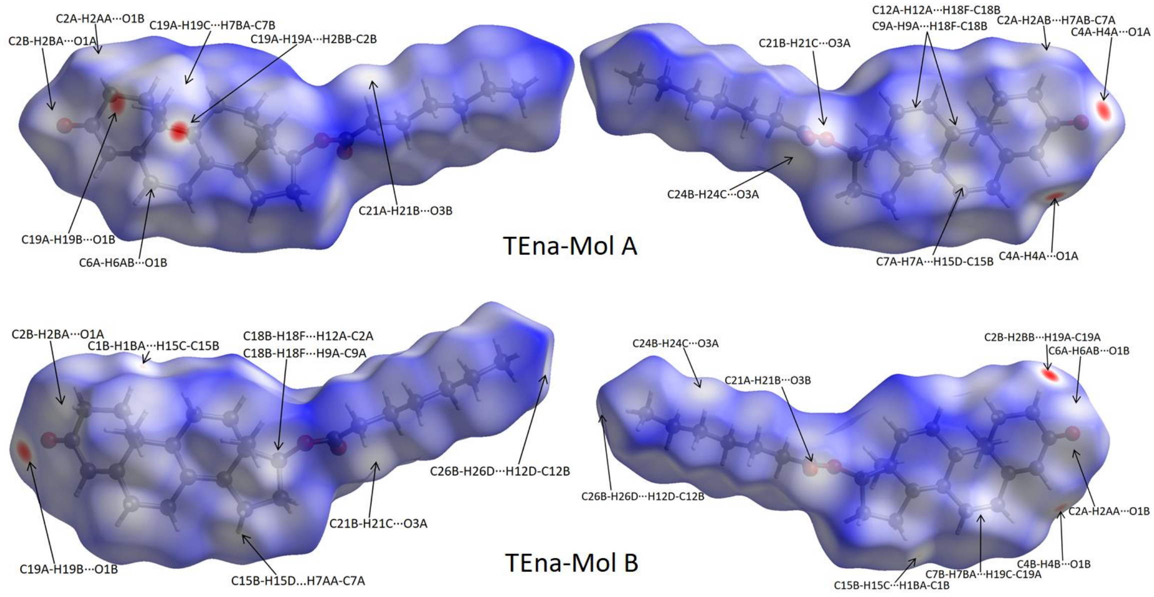
C2A-H2AA···O1B
C19A-H19C···H7BA-C7B
C2B-H2BA···O1A
C19A-H19A···H2BB-C2B
C21A-H21B···O3B
C19A-H19B···O1B
C6A-H6AB···O1B
TEna-Mol A
C12A-H12A···H18F-C18B
C9A-H9A···H18F-C18B
C2A-H2AB···H7AB-C7A
C4A-H4A···O1A
C21B-H21C···O3A
C24B-H24C···O3A
C7A-H7A···H15D-C15B
C4A-H4A···O1A
C2B-H2BA···O1A
C1B-H1BA···H15C-C15B
C18B-H18F···H12A-C2A
C18B-H18F···H9A-C9A
C26B-H26D···H12D-C12B
C21B-H21C···O3A
C19A-H19B···O1B
C15B-H15D...H7AA-C7A
TEna-Mol B
C24B-H24C···O3A
C21A-H21B···O3B
C2B-H2BB···H19A-C19A
C6A-H6AB···O1B
C26B-H26D···H12D-C12B
C2A-H2AA···O1B
C4B-H4B···O1B
C7B-H7BA···H19C-C19A
C15B-H15C···H1BA-C1B

(a)

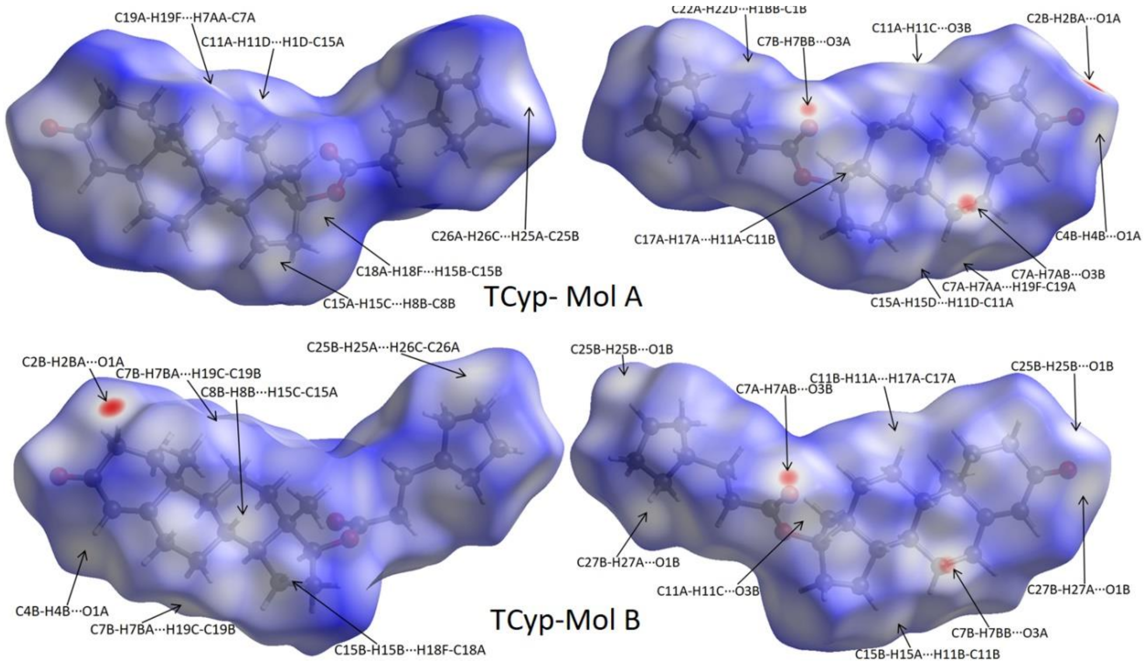
C19A-H19F···H7AA-C7A
C11A-H11D···H1D-C15A
C26A-H26C···H25A-C25B
C18A-H18F···H15B-C15B
C15A-H15C···H8B-C8B
TCyp- Mol A
C7B-H7BB···O3A
C11A-H11C···O3B
C2B-H2BA···O1A
C17A-H17A···H11A-C11B
C4B-H4B···O1A
C7A-H7AB···O3B
C7A-H7AA···H19F-C19A
C15A-H15D···H11D-C11A
C2B-H2BA···O1A
C7B-H7BA···H19C-C19B
C8B-H8B···H15C-C15A
C25B-H25A···H26C-C26A
C4B-H4B···O1A
C7B-H7BA···H19C-C19B
C15B-H15B···H18F-C18A
TCyp-Mol B
C25B-H25B···O1B
C7A-H7AB···O3B
C11B-H11A···H17A-C17A
C25B-H25B···O1B
C27B-H27A···O1B
C11A-H11C···O3B
C27B-H27A···O1B
C7B-H7BB···O3A
C15B-H15A···H11B-C11B

(b)

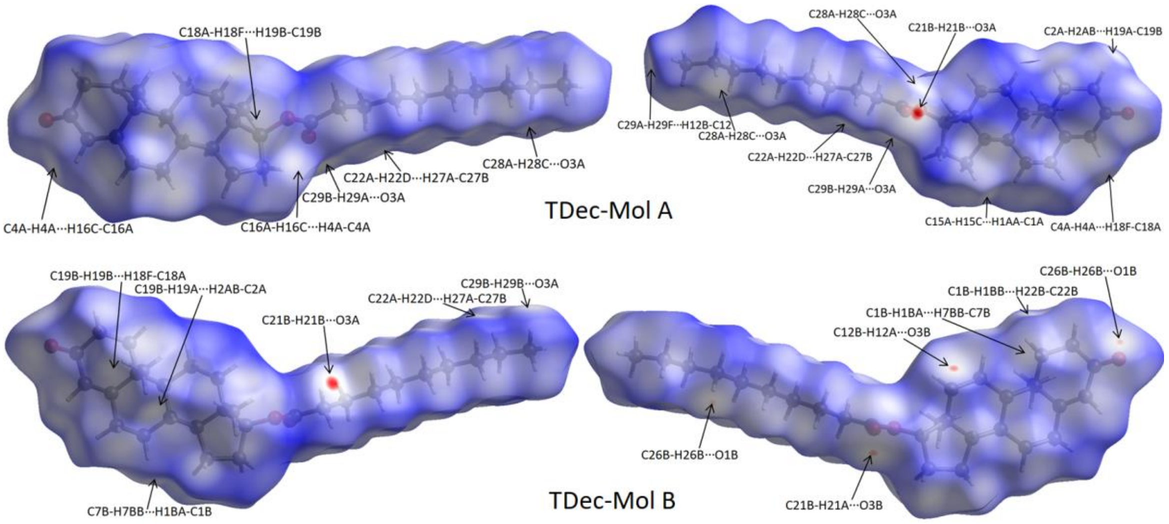

(c)

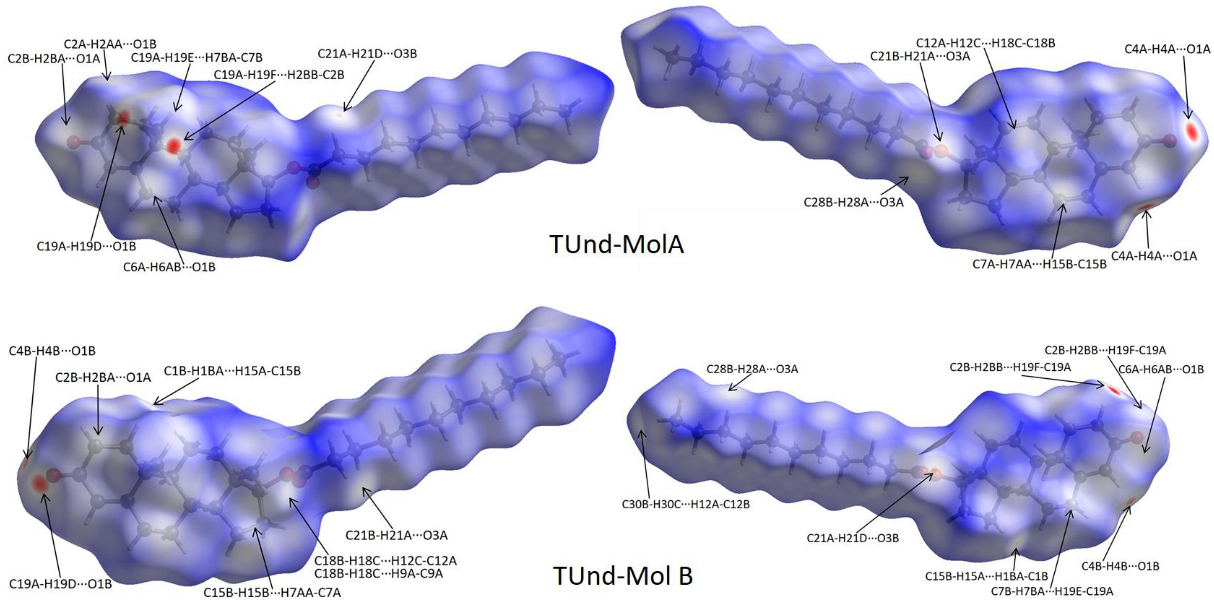

(d)

Figure S2. Hirshfeld surfaces mapped with d_{norm} ilustrating the contacts referred in Table S1. Surfaces were represented with the clour scale in the ranges as follows: TEna (a) -022 (red) to 174 (blue), -0.19 (red) to 1.93 (blue) for TCyp, -0.09 (red) to 1.68 (blue) for TDec, -0.20 (red) to 1.73 (blue) for TUnd.

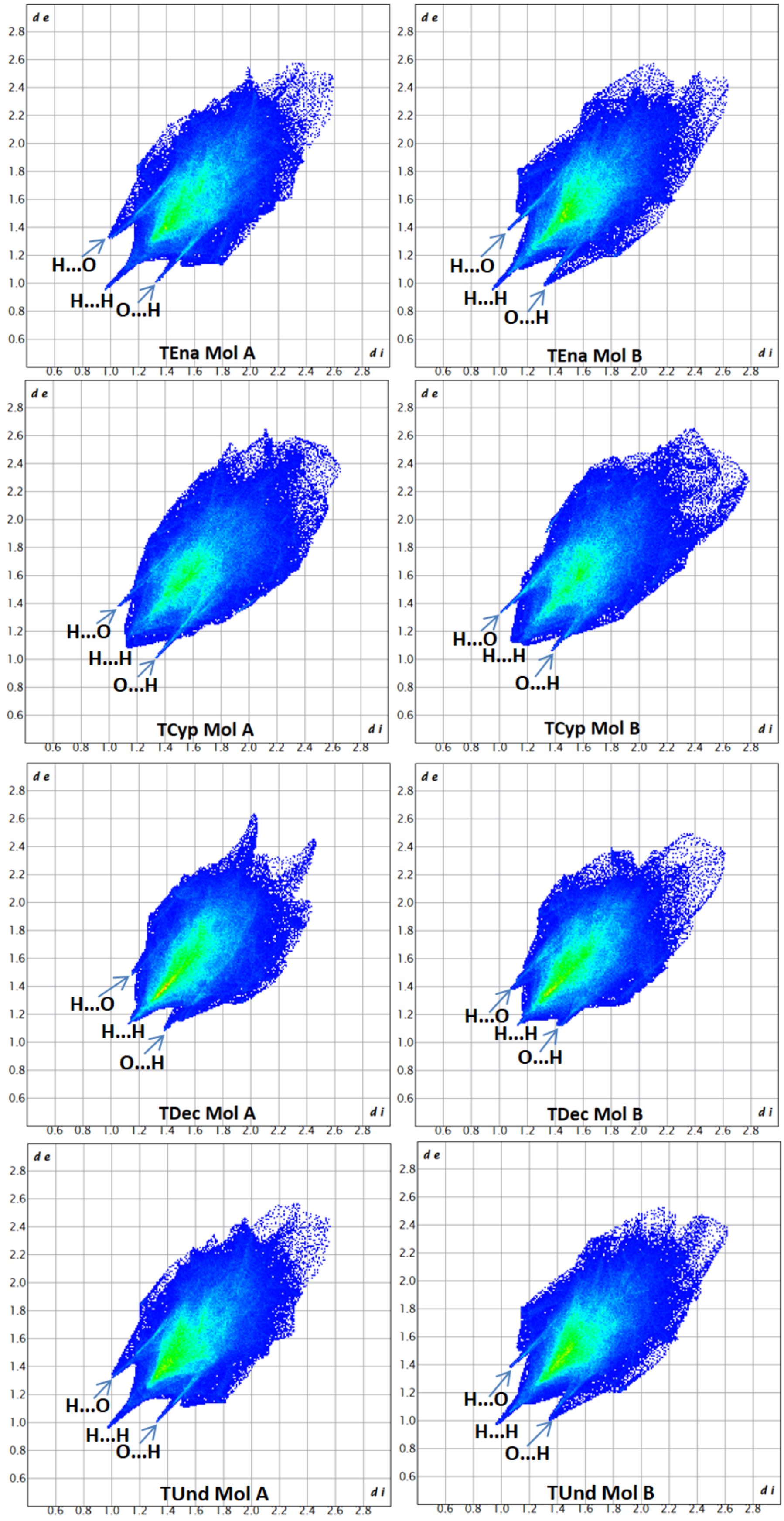

Figure S3. Fingerprint diagrams of analysed crystals

Table S1. Hydrogen bond geometry for studied crystals (Å, °)

Structure	D-H···A	D-H	H···A	D···A	<(D-H···A)
TEna	C21A-H21B···O3B	0.970	2.662(4)	3.501(5)	144.8(1)
	C19A-H19B···O1B	0,960	2.445(6)	3.321(1)	151.2(7)
	C4B-H4B···O1B	0.930	2.621(1)	3.543(2)	171.4(6)
	C4A-H4A···O1A	0.930	2.506(3)	3.414(4)	165.2(3)
TCyp	C2B-H2BA···O1A	0.970	2.449(8)	3.384(5)	161.5(7)
	C4B-H4B···O1A	0,930	2.714(1)	3.566(4)	152.7(1)
	C7A-H7AB···O3B	0.970	2.555(4)	3.485(5)	160.7(4)
	C7B-H7BB···O4A	0.970	2.583(2)	3.493(1)	156.3(3)
TDec	C21B-H21B···O3A	0.970	2.548(4)	3.305(4)	134.9(4)
	C26B-H26B···O1B	0.970	2.674(3)	3.621(1)	164.9(3)
	C12B-H12A···O3B	0.970	2.612(3)	3.358(5)	133.8(6)
TUnd	C21B-H21A···O3A	0.971	2.704(4)	3.639(6)	162.1(1)
	C21A-H21D···O3B	0.970	2.635(3)	3.447(5)	141.4(3)
	C19A-H19D···O1B	0.960	2.475(6)	3.325(4)	147.4(9)
	C4B-H4B···O1B	0.930	2.622(1)	3.548(3)	173.1(5)
	C4A-H4A···O1A	0.930	2.498(3)	3.411(2)	167.3(6)

***Table S2**. Contributions to the Hirshfeld surfaces for various intercontacts*

Structure	H···H	O···H/H···O	C···H/H···C	C···O/O···C	O···O	C···C
TEna Mol A	82.1%	14.7%	3.1%	-	-	0.2%
TEna Mol B	82.4%	14.5%	2.8%	-	-	0.2%
TCyp Mol A	82.9%	13.2%	3.7%	0.1%	-	0.2%
TCyp Mol B	79.9%	17.0%	3.0%	0.1%	-	-
TDec Mol A	84.5%	12.9%	2.6%	-	-	-
TDec Mol B	83.0%	14.2%	2.8%	-	-	-
TUnd Mol A	84.7%	12.5%	2.6%	-	-	0.2%
TUnd Mol B	85.0%	12.4%	2.4%	-	-	0.2%

Table S3. *Solubility of esters in various mixtures*

	APRICOT (mg/mL)	GSO (mg/mL)	MCT (mg/mL)	COTTON (mg/mL)	CASTOR (mg/mL)	SESAME (mg/mL)
TCyp	237.5	239.2	257	236.3	286.6	278.1
TDec	536.7	497.2	596.7	495.8	530.5	548.7
TUnd	200.1	183.1	201.8	195.5	196.3	209.1

Table S4. *Nature and magnitudes of intermolecular interaction energies for selected contacts (kJ/mol)*

Crystal	Interaction pair	Selected contact	E_{ele}	E_{pol}	E_{disp}	E_{rep}	E_{tot}
**TEna**	Molecule A-Molecule B (asym unit)	C21A-H21B-···O3B	-5.6	-3.9	-52.7	17.1	-45.1
	Molecule A-Molecule A	C4A-H4A···O1A	-9.5	-3.5	-6.6	5.0	-14.6
	Molecule A-Molecule B	C19A-H19A···H2BB-C2B	-4.8	-2.1	-27.4	11.9	-22.4
	Molecule A-Molecule B	C19A-H19B···O1B	-11.2	-3.9	-28.3	14.1	-29.3
	Molecule B-Molecule B	C4B-H4BA···O1B	-8.8	-2.5	-5.5	2.8	-14.0
	Molecule B-Molecule B	C15B-H15C···H18A-C18B	0.1	-0.4	-20.4	5.7	-14.1
**TCyp**	Molecule A-Molecule B (asym unit)	C15A-H15C···H8B-C8B<br>C18A-H18F···H15B-C15B	-1.6	-0.7	-37.0	14.3	-25.0
	Molecule A-Molecule B	C7A-H7AB···O3B	-11.6	-4.4	-47.8	13.4	-50.4
	Molecule B-Molecule A	C2B-H2BA···O1A	-13.5	-3.6	-10.5	7.3	-20.3
	Molecule B-Molecule A	C4B-H4B···O1A	-7.7	-2.8	-7.3	3.0	-14.8
	Molecule B-Molecule A	C7B-H7BB···O3A	-11.7	-3.2	-10.1	6.2	-18.8
	Molecule A-Molecule B	C26A-H26C···H25A-C25B	-12.1	-5.0	-49.3	16.1	-50.3
	Molecule B-Molecule A	C7A-H7AA···H19F-C19A	-1.6	-0.7	-41.9	12.7	-31.5
	Molecule B-Molecule A	C11A-H11C···O3B	-12.1	-5.0	-49.3	14.5	-51.9
	Molecule A-Molecule B	C15A-H15C···HH8B-C8B<br>C18A-H18F···H15B-C15B	-1.7	-0.8	-35.8	10.9	-27.4
	Molecule A-Molecule A	C19A-H19F···H7AA-C7A<br>C15A-H15D···H11D-C11A	-1.6	-0.7	-41.9	16.6	-27.6
	Molecule A-Molecule A	C15A-H15D···H11D-C11A<br>C7A-H7AA···H19F-C19A	-1.6	-0.6	-41.9	12.3	31.8
	Molecule B-Molecule B	C25B-H25B···O1B	-3.1	-1.8	-8.0	2.5	-10.4
	Molecule B-Molecule B	C27B-H27A···O1B	-4.9	-2.8	-8.0	2.4	-13.3
	Molecule B-Molecule B	C19B-H19C···H7BA-C7B	-0.9	-0.6	-41.3	12.4	-30.4
**TDec**	Molecule A-Molecule B (asym unit)	C2A-H2AB···H19-C19A	-1.8	-1.6	-25.2	5.8	-22.8
	Molecule A-Molecule A	C28A-H28C···O3A	-1.8	-0.8	-16.0	3.4	-15.2
	Molecule B-Molecule A	C29B-H29A···O3A	-3.3	-0.6	-50.9	16.0	-38.8
	Molecule B-Molecule A	C21B-H21B···O3A	-7.1	-4.2	-62.2	20.4	-53.1
	Molecule A-Molecule B	C18A-H18F···H19B-C19B	-3.8	-2.8	-32.8	10.9	-28.5
	Molecule A-Molecule A	C1A-H1AA···H15C-C1A	-2.3	-0.4	-18.5	3.3	-17.9
	Molecule A-Molecule A	C4A-H4A···H16C-C16A	-2.3	-1.4	-17.3	3.6	-17.4

	Molecule B- Molecule B	C26B-H26B···O1B<br>C12B-H12A···O3B	-9.2	-5.0	-42.4	13.0	-43.6
	Molecule B- Molecule B	C7B-H7BB···H18A-C1B	-1.3	-0.5	-20.4	5.6	-16.6
**TUnd**	Molecule A-Molecule B (asym unit)	C21B-H21A···O3A	-9.2	-3.4	-71.6	23.4	-60.8
	Molecule A-Molecule B	C28B-H28A···O3A	-4.8	-1.6	-26.8	9.9	-23.3
	Molecule A-Molecule B	C21A-H21D···O3B	-6.6	-4.2	-62.8	22.5	-51.1
	Molecule A-Molecule A	C4A-H4A···O1A	-9.4	-3.3	-6.5	5.1	-14.1
	Molecule B-Molecule B	C4B-H4B···O1B	-8.5	-2.5	-5.4	2.7	-13.7
	Molecule A-Molecule A	C29A-H19F···H2BB-C2B	-4.9	-2.1	-28.3	11.9	-23.4
	Molecule B-Molecule B	C12B-H12A···H30C-C30B	-2.9	-0.7	-54.5	15.7	-42.4
	Molecule A-Molecule B	C2B-H2BA···O1A<br>C19A-H19D···O1B	-10.8	-3.3	-27.8	13.2	-28.7
	Molecule B-Molecule B	C15B-H15A···H1BA-C1B	0.0	-0.5	-21.8	6.3	-16.0

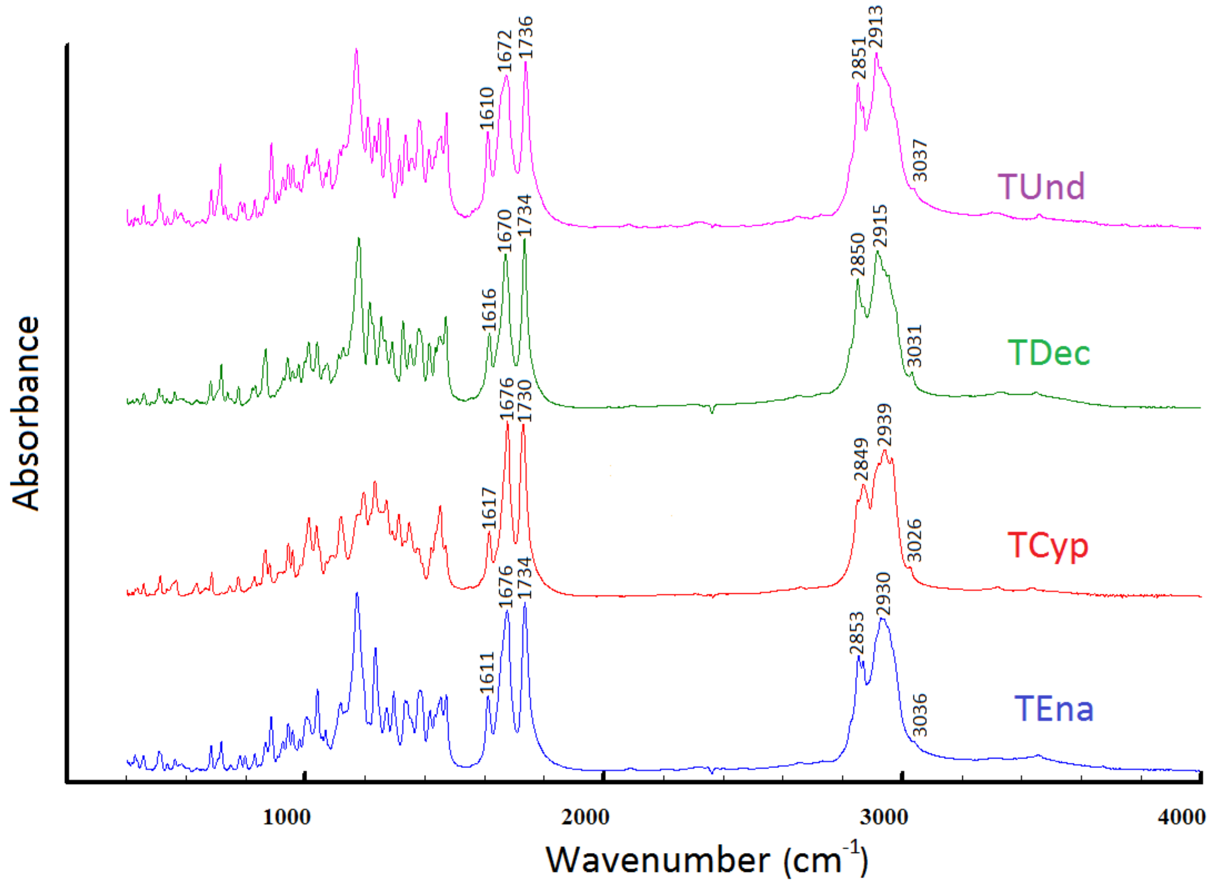

Figure S4. FT-IR spectra of analyzed esters

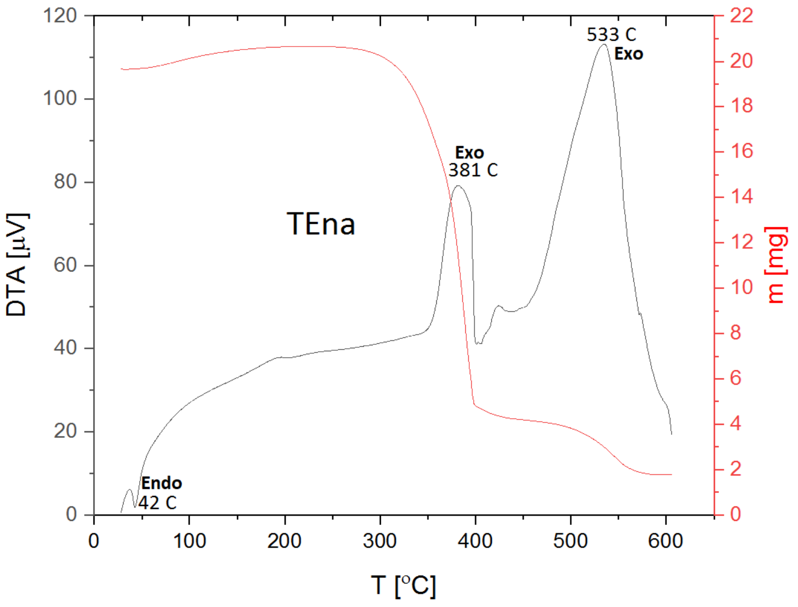

TEna
533 C
Exo
Exo
381 C
Endo
42 C
DTA [µV]
m [mg]
T [°C]

(a)

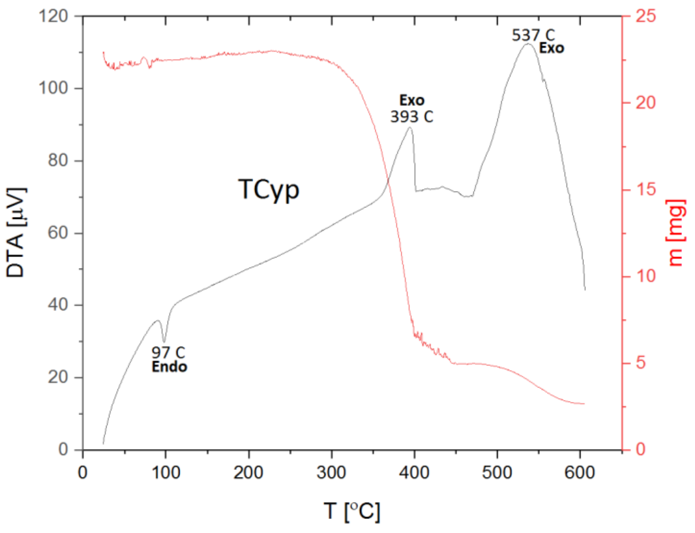

TCyp
537 C
Exo
Exo
393 C
97 C
Endo
DTA [µV]
m [mg]
T [°C]

(b)

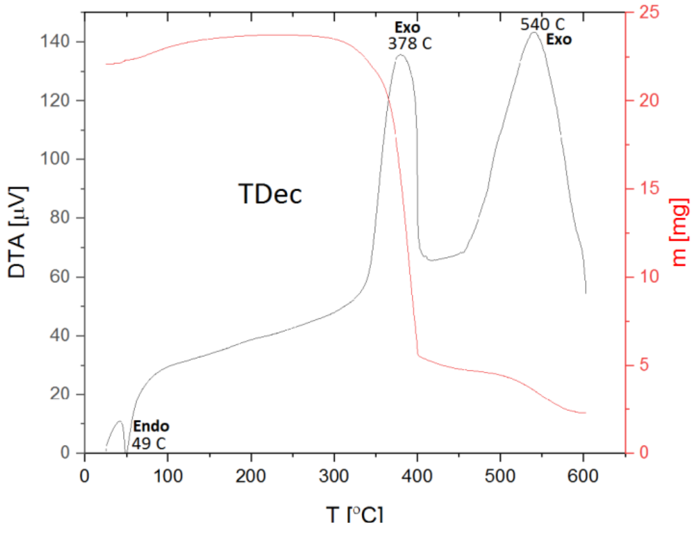

TDec
Exo
378 C
540 C
Exo
Endo
49 C
DTA [µV]
m [mg]
T [°C]

(c)

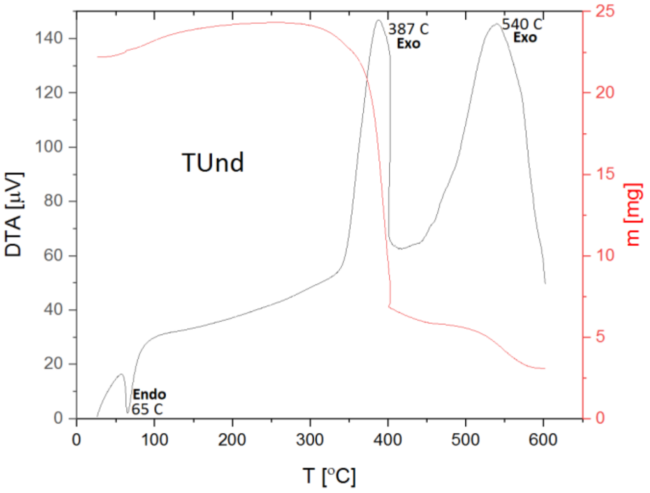

(d)

Figure S5. DTA/TG curves of analyzed compounds
